# Supplementary material for: Complexome profiling of the Chlamydomonas psb28 mutant reveals TEF5 as an early PSII assembly factor
Source: Plant Cell. 2025 Mar 17;37(6):koaf055. doi: 10.1093/plcell/koaf055 (PMC12164590; doi:10.1093/plcell/koaf055)
Supplement: koaf055_Supplementary_Data [file koaf055_supplementary_data.zip › psb28_interactive.zip!/plots/intensity/qualityPlots/peptides/_Cre02.g086850.t1.1.html]

### Cre02.g086850.t1.1

Trivial name:   
SeqCoverage [median]: 15.30  
Mass: 35.98 kDa  
  
 

| Sequence | MissedCleavage | Mass [Da] | wtIntensity | muIntensity | Score |
| --- | --- | --- | --- | --- | --- |
| EAEVLALR | 0 | 899.51 | 3166.56 | 11644.22 | 170.06 |
| LQNFVDSMASR | 0 | 1266.60 | 0.00 | 542.34 | 67.56 |
| LQNFVDSMASRR | 1 | 1422.70 | 739.53 | 760.17 | 53.24 |
| SAAAVEEGLK | 0 | 973.51 | 3524.25 | 12990.18 | 113.18 |
| SAAAVEEGLKAAK | 1 | 1243.68 | 2536.88 | 6068.16 | 99.53 |
| VLAQSPQAAAPK | 0 | 1179.66 | 0.00 | 728.95 | 118.18 |
|  |  |  | 9967.2 | 32734.0 |  |
